# Volatile versus Total Intravenous Anesthesia for Coronary Artery Bypass Graft Surgery: Analysis of 1586 MYRIAD Trial Patients Managed with the Same Perioperative Protocol

**DOI:** 10.31083/j.rcm2308265

**Published:** 2022-07-21

**Authors:** Vladimir Lomivorotov, Pavel S. Ruzankin, Rosalba Lembo, Anton S. Tarasenko, Alexander Chernyavskiy, Martina Crivellari, Fabrizio Monaco, Laura Ruggeri, Marina Pieri, Liudmila Lomivorotova, Alessandro Belletti

**Affiliations:** ^1^Department of Anesthesiology and Intensive Care, E. Meshalkin National Medical Research Center, 630055 Novosibirsk, Russia; ^2^Department of Anesthesiology anf Intensive Care, Novosibirsk State University, 630090 Novosibirsk, Russia; ^3^Sobolev Institute of Mathematics, Siberian Branch of the Russian Academy of Sciences, 630090 Novosibirsk, Russia; ^4^Department of Mathematics and Mechanics, Novosibirsk State University, 630090 Novosibirsk, Russia; ^5^Department of Anesthesia and Intensive Care, IRCCS San Raffaele Scientific Institute, 20132 Milan, Italy; ^6^Instituto Di Ricerche Farmacologiche Mario Negri IRCCS, 20156 Milan, Italy

**Keywords:** volatile anesthesia, cardiopulmonary bypass, cardioprotection, total intravenous anesthesia, cardiac anesthesia

## Abstract

**Background:**

This study investigated the influence of volatile anesthesia (VA) on major complications and mortality in patients undergoing coronary artery bypass graft surgery (CABG).

**Methods:**

This post-hoc analysis included 1586 patients from the MYRIAD trial managed using the same perioperative protocol at a single institution. Patients were randomized to receive either volatile anesthesia (sevoflurane, isoflurane, or desflurane) or total intravenous anesthesia (TIVA). The assessed study outcomes were the rate of complications, including: myocardial infarction, stroke, acute kidney injury, prolonged ventilation (>24 h), receipt of high-dose inotropic support (inotropic score >10), and need for mechanical circulatory support. The duration of intensive care unit (ICU) stay, length of hospitalization, hospital readmission during follow-up, 30-days and 1-year mortality were also analyzed.

**Results:**

1586 patients were enrolled between September 2014–September 2017 and randomly assigned to the volatile anesthesia group (n = 794) and the TIVA group (n = 792). The median patient age was 63 years, with a median ejection fraction of 60%. There were no significant differences in the rates of major complications, duration of ICU stay, and hospitalization between the groups. The median total dose of fentanyl was 12.0 mcg/kg in volatile group and 14.4 mcg/kg in TIVA group (*p *< 0.001). One-year mortality rates were 2.5% (n = 20) and 3.2% (n = 25) in the volatile and TIVA groups, respectively. Two patients were lost at the 30-day and 1-year follow-ups in the volatile group compared to four patients in TIVA group. Regression analysis showed that cardiopulmonary bypass (CPB) duration, fentanyl dose, and baseline serum creatinine level were associated with 30-days mortality, while ejection fraction was associated with 1-year mortality.

**Conclusions:**

The use of VA in patients undergoing CABG did not result in a reduction in major complications or mortality compared with TIVA. A higher dose of fentanyl was used in the TIVA group and was associated with an increase in the 30-days mortality. These findings warrant further investigation.

**Clinical Trial Registration:**

ClinicalTrials.gov (NCT02105610).

## 1. Introduction

The increase in older populations is expected to cause a subsequent increase in the number of patients with cardiovascular diseases and those undergoing cardiac surgical procedures [1]. In the USA, more than 300,000 coronary artery bypass graft (CABG) surgeries are performed each year [2]. Unfortunately, the rate of major postoperative complications following CABG procedures remains high [3, 4], despite a decrease in the 1-year mortality rate to 2–3%.

Both volatile anesthesia (VA) and total intravenous anesthesia (TIVA) for induction and maintenance of anesthesia are currently used. Numerous studies suggest that halogenated volatile agents have cardioprotective properties in patients undergoing cardiac surgery [5, 6]. According to international consensus conferences, volatile anesthetics have been identified as non-surgical interventions that reduce mortality during cardiac surgery [7, 8]. Current European and American guidelines recommend using volatile anesthetics to maintain general anesthesia during cardiac surgical procedures and cardiopulmonary bypass (CPB) [9, 10].

The MYRIAD trial was the largest multicenter randomized trial to assess the influence of volatile anesthesia on 1-year mortality in patients after CABG surgery [11]. However, the pragmatic design of the study prevented the implementation of a strict anesthetic and perioperative care management protocols, creating a potential limitation in the study. Consequently, heterogeneous clinical management may have resulted in a dilution of the cardioprotective effects of volatile agents.

This study conducted a post-hoc analysis of patients enrolled in the MYRIAD trial at a single institution, to assess whether anesthesia modalities would have a significant effect in the rate of major perioperative and postoperative complications. We hypothesized that volatile anesthesia would reduce the rate of major complications (myocardial infarction, stroke, acute kidney injury, prolonged ventilation [>24 h], receipt of high-dose inotropic support [inotropic score ≥10], need for mechanical circulatory support), duration of intensive care unit (ICU) stay, length of hospitalization, hospital readmission during follow-up, 30-days and 1-year mortality compared to intravenous anesthesia.

## 2. Materials and Methods

The MYRIAD trial (NCT02105610) is a large multicenter randomized controlled trial that assessed the influence of VA versus TIVA on 1-year mortality in patients undergoing CABG. This post-hoc analysis included 1586 patents enrolled in the MYRIAD trial at the E. Meshalkin National Medical Research Center in Russia, from the 14th of September 2014–21st of September 2017. This study was approved by the local hospital ethics committee (protocol #40; July 31, 2014), and a written informed consent was obtained from all the patients prior to enrollment. Patients aged ≥18 years who underwent elective isolated CABG surgery were eligible for the study. The exclusion criteria were unstable angina, myocardial infarction in the previous 30 days, current use of a sulfonylurea, allopurinol, or theophylline, participation in other randomized controlled trials in the previous 30 days, general anesthesia in the previous 30 days, non-elective CABG, previous kidney or liver transplantation, cirrhosis, a previous adverse response to any of the trial anesthetic agents, or a planned concomitant valve surgery or aortic surgery. The detailed protocol of the MYRIAD trial has been previously published [12].

Patients were randomly assigned in a 1:1 ratio to either VA or TIVA groups. Sealed, opaque, sequentially numbered envelopes were used for randomization. Patients, personnel who collected data, and those who assessed outcomes were blinded to the patient allocation. Owing to the study design, the attending anesthesiologists were aware of patient allocation. However, outcome assessors were blinded to the study treatment.

### 2.1 Anesthesia

Patients in the VA group received ventilation delivered sevoflurane, desflurane, or isoflurane. The patients assigned to the TIVA group received intravenous agents (propofol, benzodiazepines, and ketamine). The initial dose of propofol in the TIVA group was 4–6 mg/kg/h adjusted according to hemodynamic and clinical effects. Ketamine (0.6–0.8 mg/kg/h) or benzodiazepines (0.4–0.5 mg/kg/h) were used mainly during CPB.

Cefuroxime was administered perioperatively for infection prophylaxis. All patients received volume-controlled ventilation with an inspired oxygen fraction of 50%, tidal volume of 6–8 mL/kg, and respiration rate of 12–14 breaths/min (Primus, Evita XL; Draeger, Germany) during and after the surgery; the patients also received a positive end-expiratory pressure of 5 ${\mathrm{cmH}}_{2}$O. For invasive blood pressure monitoring, the radial artery was cannulated using a 20G catheter (Arteriofix, B Braun, Germany). The internal jugular vein was cannulated using a triple-lumen central venous catheter (Certofix; B Braun, Germany). Median sternotomy was performed in all patients. Anticoagulation with sodium heparin was used (initial dose, 3 mg/kg) to achieve an activated coagulation time >480 s during cardiopulmonary bypass (CPB). Cannulation of the ascending aorta and the right atrium was performed. A perfusion index of 2.4–2.8 L/min/$m^{2}$ during nonpulsatile CPB was applied. In patients undergoing off-pump surgery, the heart was stabilized using an octopus system [Medtronic, USA]. All surgeries were done by three experienced cardiac surgeons.

The circuit was primed with balanced crystalloid solution, mannitol, and sodium hydrocardonate. ϵ-Aminocapronic acid (20 g) was used in all patients. Ice-cold crystalloid St. Thomas solution was delivered anterogradely for cardioplegia. The mean arterial pressure during perfusion was maintained between 60–80 mmHg by bolus administration of phenylephrine and nitroglycerine. After termination of CPB, heparin was neutralized with protamine sulfate at a ratio of 1:1; no corticosteroids were administered. If the hemoglobin level decreased to <80 g/L, packed red blood cells were administered. Inotropic support (norepinephrine and epinephrine) was initiated to maintain a systolic blood pressure >90 mmHg. In these patients, the Swan-Ganz catheter was used; inotropic support was started to maintain a cardiac index of >2 L/min/$m^{2}$ after correction of hypovolemia.

Patients were transferred from the ICU to the cardiac surgery department after meeting the following criteria: fully conscious and oriented, arterial oxygen saturation >90% on room air, no episodes of severe arrhythmia, absence of bleeding, adequate diuresis (>0.5 kg/h), no need for inotropes and vasopressors, and no signs of ongoing myocardial ischemia on electrocardiography. The patients were discharged from the hospital when they demonstrated hemodynamic stability, no obvious infections, independent ambulation and feeding, normal gastrointestinal function, use of oral medications, and exercise tolerance.

### 2.2 Data Collection and Follow-Up

We collected data on the baseline and intraoperative characteristics, postoperative complications, duration of ICU stay, length of hospitalization, and mortality. Myocardial contractility was evaluated by thoracic and transesophageal echocardiography using the Simpsons method, according to institutional protocols. Patients were contacted by phone 30 days, and 1 year, after randomization to assess their vital status and record incidence of hospital readmission. In cases of loss to patient telephone follow-up, vital status assessment were requested through their respective general practitioners, contacting the city register office, or by a letter to the home address of the patient.

### 2.3 Statistical Analysis

For continuous variables, data are expressed as medians (1st quartile; 3rd quartile). For dichotomous variables, data are expressed as percentages (counts). Continuous variables were compared using Wilcoxon rank-sum test. For dichotomous variables, comparisons were analyzed using the chi-square test if all the expected values were >5, and the Fisher exact test otherwise. Statistical significance was set at *p *< 0.05.

To assess the association of preoperative and intraoperative characteristics with 1-year mortality, univariate, and multivariable logistic regression models with 1-year mortality as the outcome were used. We did not conduct a variable selection procedure but used the same variables as those selected in Landoni *et al*. [11]. We assessed the fentanyl dose/weight ratio, rather than the fentanyl dose alone, in the regression models. In addition, we added the fentanyl dose/weight ratio to the list of independent variables because a significant difference between the volatile and TIVA groups was observed for that variable.

The logistic regression models for 30-days mortality were the same as those for the 1-year mortality. In Kaplan-Meier plots, *p*-values were reported for the log-rank test.

## 3. Results

The 1586 patients scheduled for CABG, from September 2014–September 2017, were randomly assigned to receive either VA with halogenated agents or TOVA. There were no significant differences between the two groups in terms of baseline characteristics (except for a non-clinically relevant 1% difference in baseline ejection fraction) (Table 1) and intraoperative characteristics (Table 2).

**Table 1.** **Baseline characteristics**.

	Volatile	TIVA	*p*-value
	n = 794	n = 792	
Age, years	63 (58; 68)	63 (58; 68)	0.46
Sex, male	616 (77.6%)	633 (79.9%)	0.28
Weight, kg	85 (74; 95)	84 (73; 94)	0.15
EF%	60 (54; 66)	59 (53; 65)	0.043
eGFR, mL/min/1.73 $m^{2}$	71 (62; 82)	72 (63; 82)	0.58
Redo surgery, n (%)	6 (0.8%)	5 (0.6%)	0.99
Diabetes, n (%)	189 (23.8%)	220 (27.8%)	0.080
Hypertension, n (%)	725 (91.3%)	710 (89.6%)	0.30
COPD, n (%)	64 (8.1%)	50 (6.3%)	0.21
Previous vascular surgery, n (%)	192 (24.2%)	171 (21.6%)	0.24
History of MI, n (%)	521 (65.6%)	524 (66.2%)	0.86
Atrial fibrillation, n (%)	71 (9.0%)	88 (11.1%)	0.18
Preoperative medications, n (%)			
ARB or ACE inhibitors	605 (76.3%)	609 (76.9%)	0.82
Diuretics	229 (28.9%)	247 (31.2%)	0.34
Digoxin	11 (1.4%)	7 (0.9%)	0.48
Calcium-channel blockers	242 (30.5%)	224 (28.3%)	0.36
Beta-blockers	650 (82.0%)	643 (81.2%)	0.74
Beta-blockers on the day of surgery	511 (64.4%)	500 (63.1%)	0.62
Amiodarone	23 (2.9%)	24 (3.0%)	0.99
Ivabradine	4 (0.5%)	5 (0.6%)	0.75

EF, ejection fraction; eGFR, estimated glomerular filtration rate; COPD, chronic obstructive pulmonary disease; MI, myocardial infarction; ARB, angiotensin receptor blocker; ACE, angiotensin-converting enzyme.

**Table 2.** **Intraoperative characteristics**.

		Volatile	TIVA	*p*-value
		n = 794	n = 792	
CPB duration, min		55 (42; 68)	54 (44; 68)	0.54
Off-pump surgery, n (%)		144 (18.2%)	131 (16.5%)	0.43
Number of grafts		3 (2; 3)	3(2; 3)	0.95
Fentanyl, mcg/kg		12.0	14.4	<0.001
Any volatile, n (%)		788 (99.2%)	7 (0.9%)	<0.001
	Sevoflurane	715 (90.1%)	4 (0.5%)	<0.001
	Desflurane	69 (8.7%)	2 (0.3%)	<0.001
	Isoflurane	4 (0.5%)	0 (0%)	0.12
Volatile agent during CPB, n (%)		156 (19.6%)	N/A	
Crossover, n (%)		5 (0.63%)	7 (0.88%)	0.77
Intravenous anesthetics, n (%)		773 (97.5%)	792 (100%)	<0.001
	Propofol	205 (25.9%)	771 (97.5%)	<0.001
	Ketamine	130 (16.4%)	62 (7.8%)	<0.001
	Benzodiazepines	589 (74.5%)	228 (28,8%)	<0.001
Intravenous anesthetics for induction, n (%)		627 (79.5%)	792 (100%)	<0.001
	Propofol	141 (17.8%)	675 (85.2%)	<0.001
	Ketamine	117 (15.1%)	44 (5.7%)	<0.001
	Benzodiazepines	589 (74.5%)	228 (28.8%)	<0.001
Intravenous anesthetics for maintenance, n (%)		670 (84.5%)	790 (99.7%)	<0.001
Extubation in theatre, n (%)		6 (0.8%)	3 (0.4%)	0.51
Allergic reaction, n (%)		0 (0%)	1 (0.1%)	0.50

CPB, cardopulmonary bypass.

The median age of the patients was 63 (58; 68) years. The median preoperative ejection fraction was 60%. The median duration of CPB was 54 min, and the median number of grafts for a patient was three. The median total dose of fentanyl was 12.0 mcg/kg in volatile group and 14.4 mcg/kg in TIVA group (*p *< 0.001). Five patients from the volatile group and seven from the TIVA group crossed over from one group to another. In the volatile group, the most commonly used volatile anesthetic and intravenous drugs, were sevoflurane (90.1%) and propofol (97.5%), respectively. In the volatile group, 156 (19.6%) patients received volatile agents during CPB. High-dose inotropic support was required in 1.9% and 2.1% of the patients in the volatile and TIVA groups, respectively.

There were no significant differences in the rates of major complications, duration of ICU stay, and hospitalization between the groups. One-year mortality rates were 2.5% (n = 20) and 3.2% (n = 25) in the volatile and TIVA groups, respectively (Table 3). Some patients were lost at the 30-day (n = 2) and 1-year (n = 4) follow-ups.

**Table 3.** **Outcomes**.

		Volatile	TIVA	*p*-value
		n = 794	n = 792	
Mechanical ventilation in ICU, hours		5 (4; 7)	5 (4; 7)	0.59
Mechanical ventilation >24 h		6 (0.7%)	6 (0.7%)	0.99
ICU stay, days		1 (1; 2)	1 (1; 2)	0.52
High doses of inotropic support, n (%)		15 (1.9%)	17 (2.1%)	0.86
Intraaortic balloon pump, n (%)		0 (0%)	2 (0.3%)	0.25
Hospital stay, days		11 (9; 14)	11 (9; 14)	0.79
Postoperative MI, n (%)		14 (1.8%)	19 (2.4%)	0.48
Stroke, n (%)		3 (0.4%)	3 (0.4%)	0.99
Acute kidney injury, n (%)		47 (5.9%)	46 (5.8%)	0.99
	Risk	33 (4.2%)	32 (4.0%)	0.99
	Injury	13 (1.6%)	11 (1.4%)	0.84
	Failure	1 (0.1%)	3 (0.4%)	0.37
Renal replacement therapy, n (%)		4 (0.5%)	2 (0.3%)	0.69
Revision for bleeding, n (%)		7 (0.9%)	10 (1.3%)	0.62
30-day mortality, n (%)		8 (1.0%)	8 (1.0%)	0.99
1-year mortality, n (%)		20 (2.5%)	25 (3.2%)	0.53
Hospital readmission at 1-year, n (%)		78 (9.9%)	74 (9.5%)	0.82

ICU, intensive care unit; MI, myocardial infarction.

Regression analysis showed that CPB duration, fentanyl dose, and baseline serum creatinine level were associated with 30-days mortality, while ejection fraction was associated with 1-year mortality (Tables 4,5).

**Table 4.** **Multivariable regression analysis for 30-day mortality**.

	Odds ratio for multivariable model with 95% CI	*p*-value for multivariable model
Group	0.35 (0.07; 1.46)	0.16
Sex	0.47 (0.11; 2.06)	0.29
Age	1.06 (0.97; 1.17)	0.20
Weight	0.99 (0.94; 1.04)	0.80
Ejection Fraction	1.00 (0.94; 1.07)	0.94
Serum Creatinine	1.71 (0.84; 2.52)	0.012
CPB duration	1.01 (1.00; 1.03)	0.006
Fentanyl dose, mcg/kg	1.18 (0.99; 1.38)	0.047

CPB, cardiopulmonary bypass; CI, confidence interval.

**Table 5.** **Multivariable regression analysis for 1-year mortality**.

	Odds ratio for multivariable model with 95% CI	*p*-value for multivariable model
Group	0.81 (0.36; 1.77)	0.59
Sex	1.01 (0.41; 2.86)	0.98
Age	1.05 (1.00; 1.10)	0.073
Weight	1.01 (0.99; 1.04)	0.37
Ejection Fraction	0.95 (0.92; 0.98)	0.004
Serum Creatinine	1.38 (0.85; 1.99)	0.061
CPB duration	1.01 (1.00; 1.02)	0.12
Fentanyl dose, mcg/kg	1.08 (0.97; 1.20)	0.14

CPB, cardiopulmonary bypass; CI, confidence interval.

**Fig. 1.**
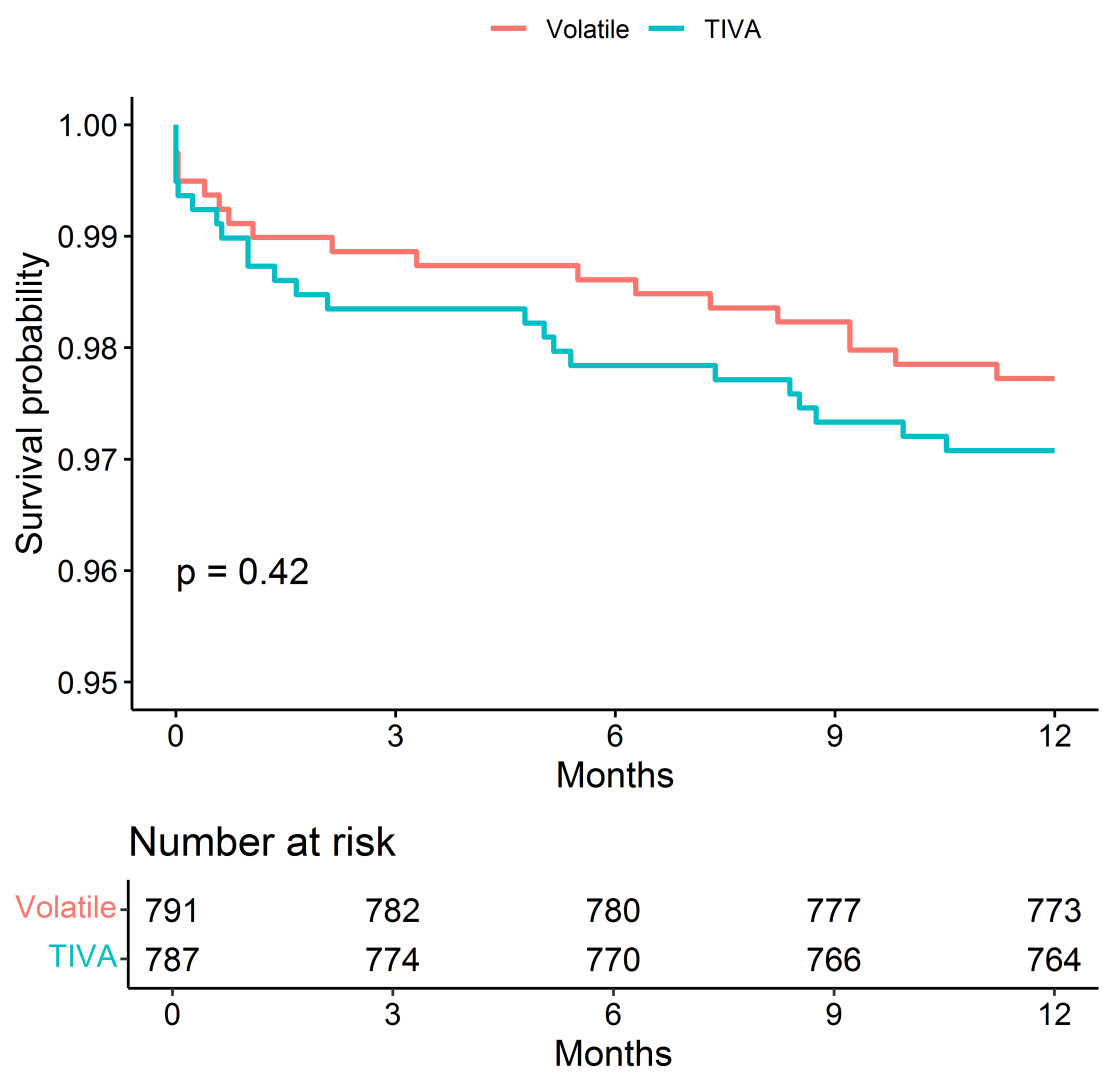
**Kaplan–Meier Survival Estimates of Death**.

Although the difference in 1-year mortality between the comparison groups was not significant, the Kaplan–Meier plot (Fig. 1) showed that mortality was lower in the volatile group.

**Fig. 2.**
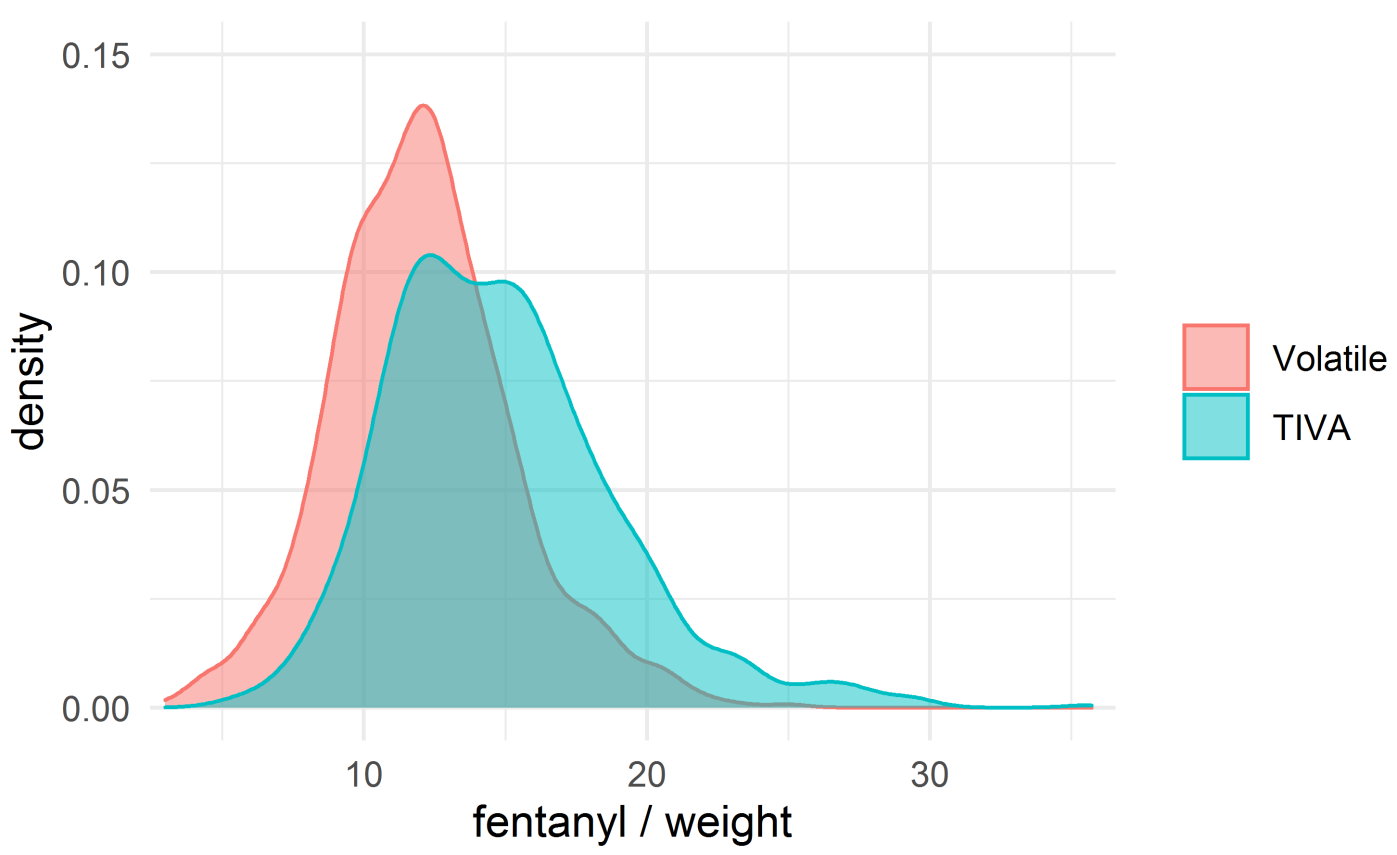
**The density plot for the dose of fentanyl (mcg/kg)**.

**Fig. 3.**
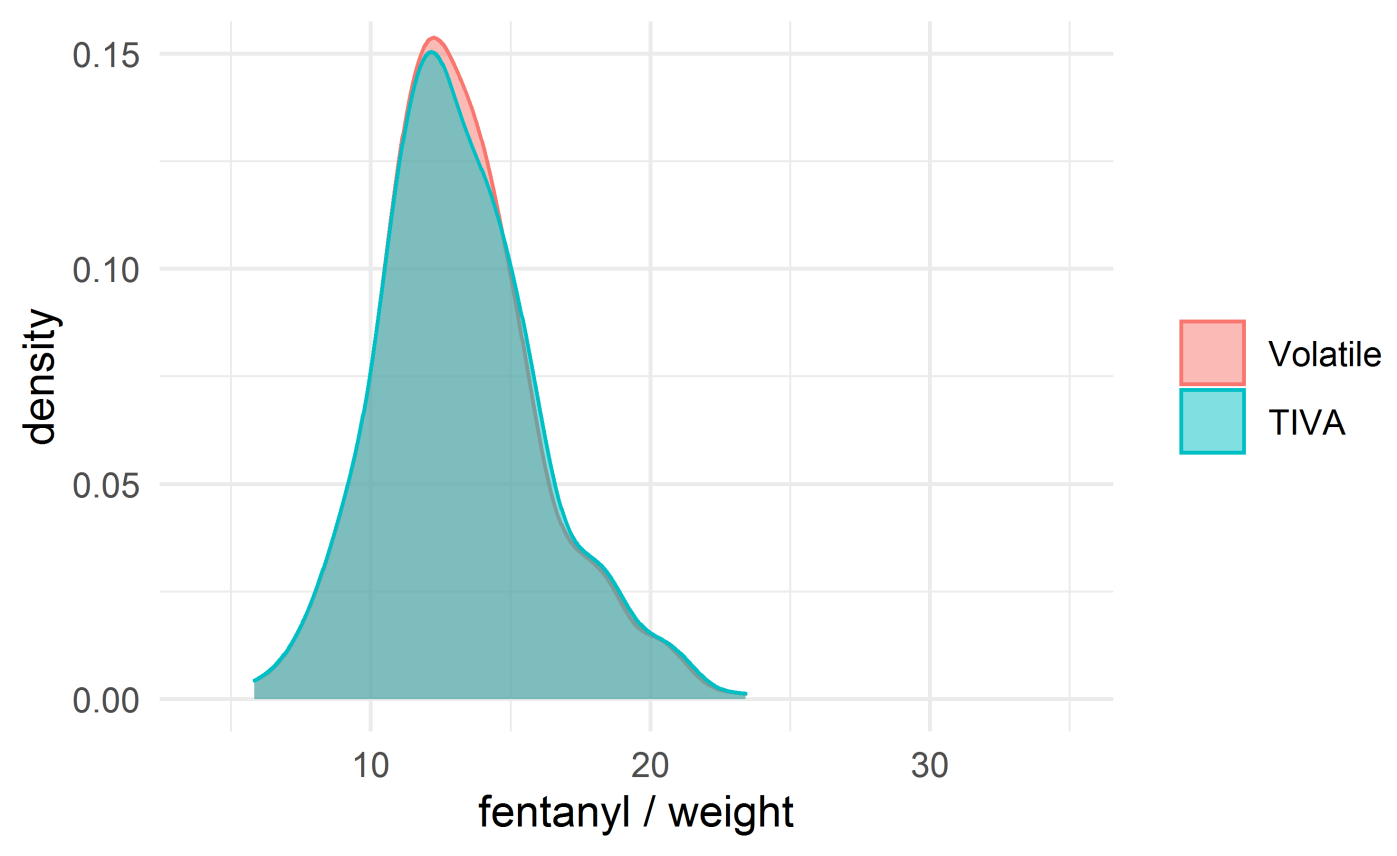
**The density plot for the dose of fentanyl (mcg/kg) after matching**.

The fentanyl dosage was a key difference between the groups; we therefore evaluated the difference in mortality after adjusting the comparison groups for fentanyl/weight, matched with a caliper of width 0.2 mcg/kg, using nearest-neighbor matching without replacement. A total of 559 matched patients were obtained in each group. The density plots for fentanyl/weight in the unmatched and matched groups are shown in Figs. 2,3.

**Fig. 4.**
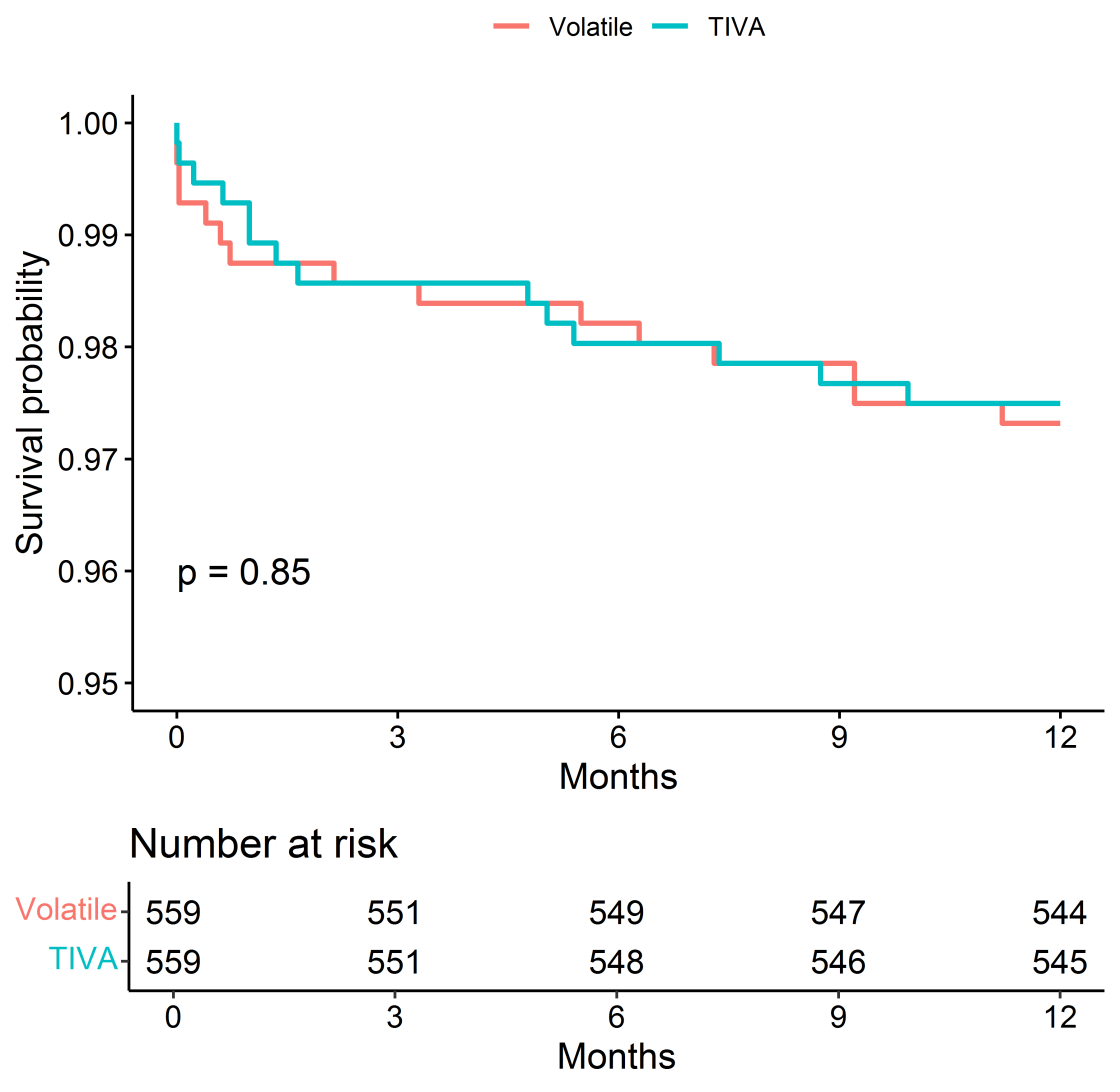
**Kaplan–Meier Survival Estimates of Death after matching the groups for the dose of fentanyl (mcg/kg)**.

The difference in 1-year mortality disappeared after matching the comparison groups on fentanyl/weight (Fig. 4).

Ejection fraction was significantly different between the two groups (*p* = 0.043, Table 1). However, the quantitative difference in median ejection fraction was 1%, which hardly corresponds to a clinically significant difference. The Cox regression model revealed that the impact of the difference on the 30-day mortality was not significant (*p* = 0.94 ).

## 4. Discussion

The findings of this study indicate that VA does not result in reduction of major patient complications, and 30-days and 1-year mortality in patients undergoing CABG surgery. Additionally, we found that fentanyl dose was associated with 30-days mortality and CPB duration was associated with 30-days and 1-year mortality.

The MYRIAD trial was the largest multicenter randomized controlled trial to assess the influence of volatile anesthesia with halogenated anesthetics on 1-year mortality in patients receiving cardiac surgery. 5400 patients were assigned to receive either VA (n = 2709) or TIVA (n = 2691). No significant differences were found between the groups with respect to mortality at the 1-year follow-up (2.8% in the VA group and 3.0% in the TIVA group) or the rate of major complications (secondary outcomes) [11].

VA is widely used in patients undergoing cardiac surgery because of its several favorable effects. Numerous randomized controlled trials have shown that VA reduces the release of myocardial injury [13, 14, 15, 16]. Our findings contrasted with the results of a large single-center randomized controlled trial by Likhvantsev *et al*. [13], which indicated a reduction in the duration of hospital stay and 1-year mortality in patients with VA. Possible mechanisms responsible for cardioprotection include opening of potassium channels, gene expression, and modulation of intracellular signaling pathways [17]. Finally, cardiac protection using VA may result in improved survival; a meta-analyses showed a reduction in mortality after CABG surgery when volatile anesthesia was used [18, 19].

Although large pragmatic multicenter studies have many advantages over single-center studies, they also carry several risks. One of the major limitations of the MYRIAD trial was that no strict protocol for perioperative care (either intraoperative or postoperative) was recommended to the participating centers. Therefore, the effect of volatile anesthetics on mortality in the findings may not be fully representative of the population due to differences in clinical management. However, our center was the top recruiter in the MYRIAD trial, and the enrolled patients were received the same protocol of perioperative care (except for the assigned trial intervention); this may have moderated the influence of differences in patient management in other study sites.

Although there was no observable clinical benefit of VA over TIVA, VA was associated with lower fentanyl consumption than TIVA. This may be attributed to the analgesic effects of sevoflurane. The definite mechanism of this effect is not well defined; nevertheless, this effect has been linked to the suppression of dorsal horn activity mainly via inhibition of excitatory postsynaptic currents in substantia gelatinosa neurons in experimental trials [20]; furthermore, the suppression of the peripheral nervous system activity may also be involved [21, 22].

The opioid-sparing effect of VA in cardiac surgery is of special importance. Until recently, high-dose parenteral opioids were the mainstay of anesthetic management for patients undergoing cardiac surgery. Nevertheless, the use of opioids is associated with numerous adverse effects, including respiratory depression and disturbance of gastrointestinal function [23]. In a large cohort of 145,735 patients undergoing non-cardiac surgery, low-dose fentanyl was linked to a lower risk of postoperative respiratory complications [24]. In cardiac surgery patients, high-dose fentanyl is associated with longer postoperative ventilation times [25]. Our findings revealed that higher fentanyl doses were associated with 30-days mortality. Therefore, multimodal analgesia to reduce the use of opioids and subsequent complications presents a promising strategy to reduce anesthesia associated complications with cardiac surgery [26]. The use of pain management drugs with different mechanisms of action (e.g., nonsteroidal anti-inflammatory drugs, pregabalin, gabapentin, ketamine, and dexmedetomidine) helps reduce the dose of fentanyl, thereby improving clinical outcomes [26]. Accordingly, the potential opioid-spring effect of VA in patients undergoing cardiac surgery may be an important component of the bundle of interventions for enhanced recovery after cardiac surgery programs, as already suggested for other clinical settings.

There were no significant observable differences in 1-year mortality due to fentanyl administration between the groups. While we can speculate that fentanyl dosage may be one of the main factors for mortality, the available sample size was insufficient to validate this hypothesis. Considering the theoretical benefits on patient outcomes from reduced opioid consumption by implementing non-opioid interventions in cardiac surgery, several studies highlight the need for future research in this area [27, 28]. Wider application of VA in cardiac surgery may magnify the observable effects of reduced fentanyl use and the subsequent 30-day mortality; however, this concept merits further investigation. Clinicians should be encouraged to further innovate viable opioid-sparing strategies in cardiac surgery, including the use of alternative analgesics and locoregional anesthetic techniques.

The propofol-based anesthesia, sevoflurane, has been reported to result in better intraoperative control of blood pressure [29, 30]. Considering that marked fluctuations in arterial blood pressure (mean duration of systolic excursion outside a range of 105–130 mmHg) is a significant predictor of 30-day mortality in patients undergoing CABG [31], the use of VA might be beneficial in reducing patient surgical risk. An anesthetic regimen including volatile agents may also be associated with a lower rate of postoperative MI and hemodynamic complications in patients undergoing CABG [32].

In this study, the CPB duration was also identified as a predictor of mortality, in corroboration with previous reports [33]. The increased duration of CPB is generally attributable to technical surgical problems that require additional perfusion time. The use of VA during CPB may potentially enhance organ protection and improve the clinical outcomes. In our study, volatile anesthetic during CPB was used in only 19.6% of the patients. In a propensity-matched study of 942 patients undergoing cardiac surgery under CPB, the administration of a volatile agent (either sevoflurane or desflurane) during CPB was associated with a reduction in troponin level after surgery as compared to propofol anesthesia [34]. Several ongoing randomized controlled trials will assess the influence of VA administration (including CPB) on the clinical outcomes. Some studies have shown that anesthesia with volatile agents provides better cerebral protection than TIVA [35]. DELICATE (Delirium Reduction by Volatile Anesthesia in Cardiac Surgery) trial is a large RCT that will enroll 672 patients and will test the hypothesis that total VA will be associated with reduction of delirium in patients >65 years of age who underwent CPB for comparison with TIVA (NCT03729011). The pilot COPIA study will assess the feasibility of a large trial on the influence of total volatile anesthesia (administered during CPB as well) versus total intravenous anesthesia on survival (NCT04039854).

Our study has several strengths. First, a uniform perioperative management protocol was used for all patients enrolled in the study. Second, three experienced cardiac surgeons performed all surgeries, which may have reduced the influence of varying surgical techniques on clinical outcomes. Third, the vital status at the 1-year follow-up was obtained for almost all patients.

Our study has several limitations. As troponin was not routinely evaluated in our patients, we could not determine the influence of the anesthesia technique on cardioprotection. Owing to the design of our study (post-hoc analysis), the required sample size was not estimated. It is possible that the risk of 1-year mortality in our study population was either underestimated or overestimated. However, the sample size calculation was performed based on mortality reported in several high-quality trials. Indeed, the overall mortality in the MYRIAD study was perfectly in line with the expected rate (2.9% vs. 3.0%) [11]. In terms of perioperative ischemic events, the overall rate of myocardial infarction (2.4%) was in line with data from previous trials on volatile anesthetics in cardiac surgery [14]. Therefore, we believe that our study population is representative of the overall global population undergoing CABG and its perioperative risks. We didn’t collect the data on complete revascularization in two groups. Presence of unrevascularized areas of myocardium might have influenced results of the study.

We cannot exclude the possibility that patients with higher risks may benefit more from VA; however, previous trials and subgroup analyses of the MYRIAD study do not support these findings [11, 14, 36]. It is possible that data from a recently completed VISION study [37] may provide clarification on risk factors for perioperative ischemic cardiovascular adverse events, facilitating identification of patients who may benefit from VA over TIVA.

## 5. Conclusions

In conclusion, the use of VA in patients undergoing CABG did not result in a reduction in major complications or mortality compared with TIVA. A higher dose of fentanyl was used in the TIVA group and was associated with an increase in the 30-days mortality. These findings warrant further investigation to develop a more informed operative anesthesia selection protocol.

## Abbreviations

CABG, coronary artery bypass graft surgery; VA, volatile anesthesia; TIVA, total intravenous anesthesia; CPB, cardiopulmonary bypass; ICU, intensive care unit.
